# Where Environment Meets Cognition: A Focus on Two Developmental Intellectual Disability Disorders

**DOI:** 10.1155/2016/4235898

**Published:** 2016-07-28

**Authors:** I. De Toma, L. Manubens-Gil, S. Ossowski, M. Dierssen

**Affiliations:** ^1^Cellular and Systems Neurobiology, Systems Biology Program, Centre for Genomic Regulation, The Barcelona Institute of Science and Technology, 08003 Barcelona, Spain; ^2^Pompeu Fabra University, 08003 Barcelona, Spain; ^3^Genomic and Epigenomic Variation in Disease Group, Centre for Genomic Regulation (CRG), The Barcelona Institute of Science and Technology, 08003 Barcelona, Spain

## Abstract

One of the most challenging questions in neuroscience is to dissect how learning and memory, the foundational pillars of cognition, are grounded in stable, yet plastic, gene expression states. All known epigenetic mechanisms such as DNA methylation and hydroxymethylation, histone modifications, chromatin remodelling, and noncoding RNAs regulate brain gene expression, both during neurodevelopment and in the adult brain in processes related to cognition. On the other hand, alterations in the various components of the epigenetic machinery have been linked to well-known causes of intellectual disability disorders (IDDs). Two examples are Down Syndrome (DS) and Fragile X Syndrome (FXS), where global and local epigenetic alterations lead to impairments in synaptic plasticity, memory, and learning. Since epigenetic modifications are reversible, it is theoretically possible to use epigenetic drugs as cognitive enhancers for the treatment of IDDs. Epigenetic treatments act in a context specific manner, targeting different regions based on cell and state specific chromatin accessibility, facilitating the establishment of the lost balance. Here, we discuss epigenetic studies of IDDs, focusing on DS and FXS, and the use of epidrugs in combinatorial therapies for IDDs.

## 1. Epigenetics and Cognition 

Intellectual disability disorders (IDDs) are complex multifactorial illnesses involving chronic alterations in neural circuit structure and function as well as likely abnormalities in glial cells. Converging evidence indicates that epigenetic control of gene expression is pivotal to learning and memory, as underscored also by the range of intellectual disabilities and behavioural deficits increasingly traced to a staggering number of epigenetic modulators. This review focuses on the importance of epigenomics in neuroscience, especially in neurodevelopment and cognition. Since epigenetic mechanisms are reversible, they are targets of interest in conceiving new therapies for the treatment of IDDs. We will specifically address two genetic intellectual disabilities, Down Syndrome (DS), caused by trisomy 21 [1], and Fragile X Syndrome (FXS), caused by the absence of FMRP protein upon a “CGG” triplet expansion at the 5′-UTR of the FMR1 gene [2]. Both IDDs show epigenetic dysregulation and, despite the differences in their neuropathological signs, share disturbances in the molecular events that regulate the way nerve cells develop dendritic spines.

### 1.1. Epigenetic Mechanisms Regulate Neurodevelopment and Cognition

**Figure 1:**
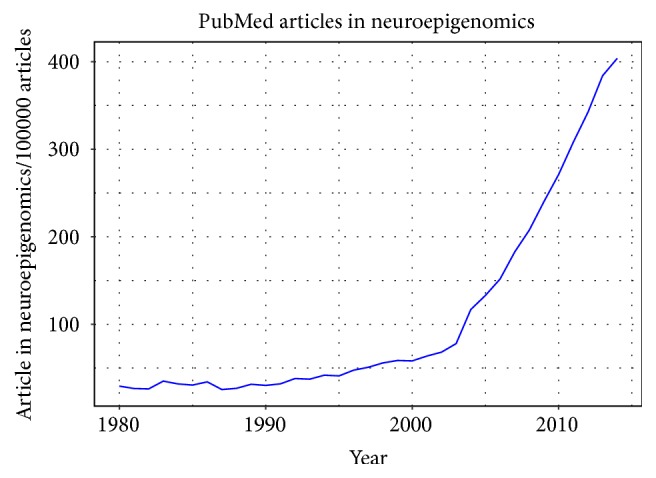
Trends in publications in the field of neuroepigenetics. The plot shows the number of publications on* PubMed* by year, normalized by the total of number of articles. The *x*-axis represents the years, and the *y*-axis plots the number of articles in neuroepigenetics per 100.000 articles.

Since the first definition of epigenetics [3] the meaning of this term has broadened to include several mechanisms of gene expression regulation not interfering with the DNA sequence but regulating the chromatin state. These include DNA chemical modifications, histone posttranslational modifications, chromatin remodelling, and the expression of noncoding RNAs (ncRNAs). Even though these mechanisms are quite different, they have in common interfering with chromatin compaction. Nuclear proteins and DNA compose chromatin that can be more condensed impairing transcription, or more loose, facilitating gene expression. The notion that experience modulates cognitive function and development has become an accepted tenet of modern neuroscience. However, the precise molecular mechanisms by which the environment modulates neurological development are still to be elucidated. One such mechanism is cognitive-activity-dependent gene expression [4]. Epigenetics mediates the interaction between the environment and the genome and, therefore, epigenetic control of gene expression is pivotal to learning and memory and can explain brain plasticity, the capacity of neurons to remodel their structures based on external inputs. This is important for two well-studied aspects in neuroscience: neurodevelopment and cognition (e.g., memory and learning), two components that are somehow interconnected as highlighted by the common mechanisms that underlie developmental and adult experience/learning associated synapse addition. In neurodevelopmental disorders such DS or FXS, problems in neural development come along with the adult cognitive impairment [1] but while dendritic spine numbers are lower and dendritic tree is affected in DS [5], FXS appears to be the only form of intellectual disability that exhibit increased numbers of dendritic spines without alterations in the dendritic arbour [6]. Recent studies established that neuronal activity triggers local de novo synthesis of proteins in the dendrites of the affected postsynaptic neurons, and the concept of a dynamic proteome at the synapse is beginning to emerge [7]. In fact, the number of papers dealing with both epigenetics and neuroscience has started to grow steadily especially after the establishment of next-generation sequencing techniques in 2004, reaching over 400 publications every 100,000 on PubMed (Figure 1). This has led to the definition of a new emerging field termed “neuroepigenetics” [8] or “neuroepigenomics” [9]. Since epigenetic mechanisms are important regulators in both neurodevelopment and cognition, we believe that these neuroepigenomics studies will be crucial in understanding the pathogenesis of neurodevelopmental IDDs, where both defects in brain development and cognition coexist. This review collects recent evidence confirming this hypothesis, pointing out how tackling epigenetic deregulation could be an ideal therapeutic approach for restoring the phenotype in neurodevelopmental IDDs.

#### 1.1.1. Chemical Modifications of DNA

The family of enzymes called DNA methyltransferases (DNMTs) catalyse the most studied modification of DNA. DNMTs transfer a methyl group from S-adenyl methionine (SAM) to a cytosine residue to form 5-methyl-cytosine (5mC). Cytosine methylation occurs especially at CG dinucleotides (CpG sites) which are underrepresented in the genome since 5mC tends to deaminate into thymine [10]. Those sites are usually methylated with the exception of CpG islands, ≈0.2–1 kb conserved regions with higher density (>50%) of CpG sites, which are usually found on gene promoters [11]. Generally speaking, this modification represses transcription both by sterically interfering with transcription factor binding and especially by recruiting repressive complexes upon binding to proteins with methyl binding domains [12].

In mammals, three main DNMTs exist: DNMT1 is called the “maintenance DNMT” since it usually binds to hemimethylated sites avoiding passive demethylation during DNA synthesis and DNMT3a and DNMT3b are the so-called de novo DNMTs [13]. Interestingly, DNMTs are highly expressed in the brain not only during neurodevelopment but also in postmitotic neurons [14], suggesting a role for DNA methylation beyond development, which is connected to brain functions in the adult. As a matter of fact, although DNA methylation has been thought to be a static epigenetic mark that could be lost only by passive demethylation during cell division, nowadays it is known that DNA methylation is dynamic and can be also actively regulated. TET enzymes initially oxidize 5mC, and, in a second phase, it can be deaminated by AID/Apobec enzymes or further TET-oxidized. Finally, the oxidation products are repaired by the base excision repair (BER) [15, 16].

Several studies highlight regulation by DNA methylation at the promoters of key genes involved in cognition. Interestingly, following contextual fear conditioning, one of the most used models for studying memory in rodent models, DNMTs are upregulated in the hippocampus during memory formation and this results in an increase in DNA methylation at the promoter of the memory suppressor gene PP1 and a decrease in the methylation at the promoter of the synaptic plasticity gene RELN during memory consolidation. Accordingly, inhibition of DNMTs resulted in PP1 demethylation and problems in memory consolidation [17]. The same is true for the BDNF gene, where DNA methylation regulation upon the learning task results in the specific increase in BDNF exons I and IV mRNA transcript during consolidation of fear memory [18]. Those changes in DNA methylation were dynamic, acute (40 minutes), and transient, being reverted in 24 hours. This finally contradicts the dogma depicting DNA methylation as a static mark and supports the hippocampus' role in memory formation and consolidation.

Moreover, the brain shows particularly high levels of two other methylation types: non-CpG methylation (mCH, where H stands for adenine A, thymine T, or cytosine C) and hydroxymethylation (5hmC), suggesting a specific neural role for these modifications [19, 20]. While mCH is absent in the foetal cortex, it accumulates in neurons during early postnatal development becoming the main form of DNA methylation and repressing critical genes during development. In this context DNMT3a seems to play a critical role. Of note, neurons show higher mCH levels than glial cells, but neuron-specific genes are repressed and methylated at the level of CH in glial cells [21]. As regards hydroxymethylation, recent studies suggest that 5hmC is not a simple intermediate product in the oxidative cytosine demethylation pathway as it was initially thought, but it is involved in keeping gene promoters ready for gene activation, preventing their DNA methylation. In agreement with this, TET1 overexpression resulted in impaired contextual fear conditioning during memory formation [22], while TET1 knockout results in defects in memory extinction and synaptic plasticity [23].

Heyward and Sweatt [51] proposed a very appealing model according to which in basal state conditions memory promoting genes are methylated and kept silenced while memory suppressor genes are basally expressed. Upon learning, both TET proteins and DNMTs are induced, the former derepressing memory promoting genes and the latter silencing memory suppressor genes. After sufficient time, the basal state is restored probably through TET-mediated derepression of memory suppressor genes and DNMT remethylation of memory promoting genes. However, what mechanisms give rise to the basal state differences in gene promoter methylation is still not known.

But how can this transient mark lead to memory storage, where memories can last a lifetime? There should be a self-perpetuating mechanism. Many studies on DNA methylation investigated the hippocampal role in memory formation and consolidation but not the further consolidation of this information in remote memory. According to a well established model, bursts of activity called “sharp-waves” would promote cortical plasticity, transferring memories from the hippocampus to the neocortex [52]. Heyward and Sweatt speculate that these waves would result in the epigenetic storing of the learning event in cortical cells, probably through double strand DNA methylation, which would be highly resistant to erasure thanks to the self-perpetuating action of DNMT1, which recognizes the hemimethylated helix and methylates the unmethylated strand [51]. Supporting the role of DNA methylation in maintaining memories, the CaN (calcineurin) gene showed delayed (1 day) and persistent (>30 days) DNA methylation in cortical neurons upon contextual fear memory even after protein levels returned to baseline, during the process of transition of contextual fear memory from “transient” (hippocampus) to “remote” (prefrontal cortex) [24].

#### 1.1.2. Histone Modifications

Histones are the main protein component of the chromatin and come in 4 flavours: H2A, H2B, H3, and H4. These basic proteins strongly associate with the DNA forming an octamer called nucleosome, along which 147 bp of the DNA helix wrap around. Additional compaction is performed by the H1 linker histone, which binds the nucleosome at its entry and exit site. Importantly, long protruding tails depart from each histone core and their posttranslational modifications (PTMs) regulate the level of chromatin compaction [53]. There are several PMTs acting on histone tails such as acetylation, methylation, phosphorylation, SUMOylation, and ADP-ribosylation. However, we only need a minimal set of epigenomic features to define chromatin states and most studies focus on specific and recurrent histone modifications [54].

Histone acetylation has a positive effect on transcription by relaxing the chromatin compaction. The acetyl group neutralizes the positive charges on Lysine (K) and Arginine (R) residues, decreasing the electrostatic interactions between the nucleosome and the DNA. The writers of this epigenetic modification are called histone acetyl transferases (HATs), while the erasers are called histone deacetylases (HDACs) [27].

Histone acetylation has emerged as a key mechanism of memory regulation. One of the first studies showed how novel tastes induce long-lasting Lysine acetylation through ERK/MAP pathway activation in the insular cortex [55]; the same was true for contextual fear conditioning during memory formation [56]. Several subsequent studies showed that global HDACs inhibitors (HDADi) improve cognitive impairments and boost learning and memory [27]. Acetylation occurs in several K residues such as H3K9/14/27 and H4K12 but also in H2B and many other sites. According to the current view, these modifications play an important role in establishing a permissive transcription, preparing cells to activate gene expression upon specific stimuli [57]. Even though it was initially thought that HDAC inhibitors enhanced gene expression globally and nonspecifically, it is now clear that specific molecules, such as the CREB transcription factor, regulate their action. CREB recruits the coactivator CBP that through its HAT domain increases acetylation at the level of the genes involved in memory consolidation [28].

Several HDAC isoforms can regulate histone acetylation levels in the adult brain. For instance, while HDAC5 is important in the nucleus accumbens, the reward centre of the brain and its disruption result in a hypersensitive response to chronic drug abuse [30]; HDAC2 was found to negatively deregulate memory formation and synaptic plasticity [58], and HDAC3 inhibition enhanced long-term object memory formation [29]. While, generally speaking, the effect of HDAC inhibition is positive for cognitive activities, this is not the case for the sirtuin family of HDACs, where SIRT1 obliteration impairs hippocampal memory formation, a defect that can be explained by decreased dendritic branching and spines, which are specialized structure for cognition [59]. Subsequent studies showed also how HDCA1 is required for fear extinction learning through a mechanism involving H3K9 deacetylation [31] and HDAC4 is required for synaptic plasticity and memory formation [60].

Histone acetylation often correlates with histone phosphorylation. For example, H3 phosphorylation at serine (S) 10 (H3S10P) together with acetylation of H3K9 is induced during spatial memory formation and facilitates the early gene activation (c-Fos, Erg1, and Arc) of the ERK/MAPK pathway [61].

The second most studied histone modification is methylation. While histone acetylation always results in transcriptional activation, histone methylation effects depend on the protein complexes docking on the different modifications. For example, H3K4 methylation and monomethylation of H3K9 (H3K9me1) result in transcriptional activation, whereas H3K9me2 and H3K9me3 result in transcriptional silencing. Histone methylation can occur at either Lysine (K) or Arginine (R) and is performed by a group of proteins containing SET domains called histone methyl transferases (HMTs). Despite being conceived initially as a static histone modification, whose half-life coincides with the histone turn over itself, histone methylation has shown to be dynamically regulated through the action of histone demethylases (HDMs) such as LSD1 for H3K4me and H3K4me2 and JMJD1a for H3K9me and H3K9me2 [62].

H3K4me3 is usually present in the proximity of the transcription start site of active genes and it has been shown to be induced one hour after contextual fear conditioning, activating promoter regions of memory genes such as ZIF268 and BDNF, to return to baseline levels at 24 hours, underlining a role in memory formation. A similar dynamic was observed for the transcriptional repressive H3K9me2 mark. Interestingly, mice deficient in* Mll*, a H3K4 methyltransferase, show defects in contextual fear memory formation [34]. In parallel, GLP/G9a, an H3K9me2 methyltransferase, is extremely important for cognition. H3K9me2 is a “switching chromatin signal” [63], acting during both development and cognition and modulating gene expression by recruiting reader, writer, and eraser enzymes. This complex is required during memory consolidation both in the hippocampus and in the entorhinal cortex [35]. Moreover, H3K9me2 is induced from 1 hour up to 25 hours upon fear conditioning, and fear memory is enhanced when inhibiting both its demethylation (LSD1-mediated) and its methylation (GLP/G9a-mediated) [64]. Finally, GLP/G9a is also important in adaptive behaviour since its deficiency leads to defects in learning, motivation, and environmental adaptation [37].

Although less studied, several other histone methylation marks play an important role during cognitive processes. For example, H3K36me3, marking the 3′ end of transcribed genes, is immediately induced during object recognition memory in both the hippocampus and prefrontal cortex and is reactivated after activation of recent (24 h) and remote (7 days) memory, with hypermethylation of the ZIF268 promoter [36].

Future research should focus on integrative analysis showing how those marks crosstalk and what is the precise dynamic of their activation.

#### 1.1.3. Chromatin Remodelling

Nucleosome remodelling complexes (NRCs) alter nucleosome positioning in an ATP-dependent way, promoting nucleosome sliding, eviction, or histone variants exchange. In the brain the most studied NRC is the neuron-specific Brg1/hBrm Associated Factor (nBAF) complex, a multiprotein complex belonging to the SWI/SNF family that regulates gene expression in both development and adult cognition. Of particular importance in neurodevelopment is the upregulation of the BAF45b and BAF45c subunits and the switch between the BAF53a and BAF53b, which begins at E12.5 and is exclusive to postmitotic neurons, being essential for BRG1's ATPase activity [65]. This complex has shown to be important in cognition since BAF53b deficient mice showed large impairments in long-term memory formation [46].

#### 1.1.4. Noncoding RNAs (ncRNAs)

Noncoding RNAs (ncRNAs) are transcripts that are not translated into a protein. They include two broad categories: small RNAs and long noncoding RNAs (lncRNAs). The first comprehends micro-RNAs (miRNAs) that generally inhibit gene expression by complementarity to their targets and PIWI interacting RNAs (piRNAs), involved in transposon repression through RNA mediated DNA methylation. The function of long noncoding RNAs is less known; while initially thought to be “transcriptional noise,” recent studies suggest that lncRNAs can regulate gene expression by acting as “guide” or scaffold RNAs, targeting epigenetic changes to specific genomic locations. Many noncoding RNAs have been identified in the brain, and approximately 40% of them are not found in other tissues [66]. While for most of the lncRNA the mechanism remains elusive, extensive evidences suggest they have important roles in neural development, synaptogenesis, and synaptic plasticity [67]. Notably, TUNA, RMST, and DALI regulate neural differentiation by directing transcription factors, chromatin-remodelling machineries, and DNMTs to important genomic loci [41, 68, 69].

The complex picture of epigenetic regulation in the brain can be puzzling, with some epigenetic changes enhancing cognition and other impairing neural activities. However the take-home message is that every kind of epigenetic change has been found associated with neural activity, indicating that a correct balance of the epigenetic machinery is needed for a proper neural function. Moreover, epigenetic changes should not be seen as distinct and isolated events. Repressive modifications tend to occur together and the same is true for permissive modifications. As an example, several methyl binding proteins recruit HDACs allowing cytosine methylation and histone deacetylation to act in concert to repress gene transcription [70]. That means that epigenetic mechanisms orchestrate the specific gene expression activated upon brain activity.

## 2. Epigenetic Dysregulation in Intellectual Disabilities

Many intellectual disability disorders arise from mutations affecting the function of the epigenetic regulators discussed in Section 1, underlining the importance of a correct balance between readers and erasers of epigenetic modification for a proper brain function (Table 1).

Besides the epigenetic syndromes arising by direct perturbation in the functions of key epigenetic molecules, several, if not all, other syndromes and IDDs have probably an epigenetic component or origin. Epigenetics means dynamics and reversibility, and thus a lack of epigenetic coordination may lead to defects in neurodevelopment with consequent defects in cognition. In this context it is obvious that an early therapeutic intervention is preferential, but given the reversibility of epigenetic processes, it is in theory possible to restore a proper neuronal function ameliorating the cognitive impairment in this developmental IDDs. To what extent this is feasible, together with the efficacy and duration of the effect for this approach, remains to be elucidated. The best therapeutic strategies will likely consist in combinatorial therapies using both neuromodulators and “epidrugs” as cognitive enhancers.

In the end of the section we will specifically focus on two developmental genetic disorders, DS and FXS, showing how both development and cognition are interconnected and how epigenetic regulation is essential in both processes as a gateway for processing inputs received from the environment.

### 2.1. Cognitive Function, Synaptic Plasticity, and Epigenetics

Epigenetic effectors involved in intellectual disability developmental disorders are likely interacting with fundamental players in neuronal maturation. For instance, nBAF complexes regulate genes essential for dendritic outgrowth and spine formation [71], and the activity of GLP/G9A, MeCP2, and ncRNAs affects the regulation of BDNF expression whose role in neuritogenesis, synaptogenesis, spine maturation, and axonal arborisation has been thoroughly assessed and is reviewed elsewhere [72, 73]. BDNF is of specific importance for initiating guided branching in the cell membrane [74] and for completing spine maturation [75].

Converging evidence indicates that epigenetic control of gene expression is also pivotal to learning and memory through its crosstalk with neuronal activity and synaptic plasticity mechanisms, as underscored also by the range of intellectual disabilities and behavioural deficits increasingly traced to a staggering number of epigenetic modulators. Specifically, several epigenetic modifications act as key signalling relay in the integration of synaptic inputs, as vividly shown for histone acetylation in CREB-dependent changes triggered during NMDA-receptor mediated long-term potentiation (LTP) [76], but also more recently for the rapid surges of DNA methylation and demethylation and 5-hydroxymethylation in response to neuronal activity [21]. Some epigenetic marks, including DNA methylation and histone methylation on Lysine 9 and Lysine 27 of histone H3, can be stably propagated over extended periods of time, in proliferating and postmitotic cells, implying alternative neuronal activity-dependent plasticity mechanisms putatively involved in learning and memory. Through recruitment mechanisms still poorly understood, epigenetic modifiers can exert genome-wide but also highly gene-specific effects. These features have made epigenetics the focal point of the grounding in molecular terms of how Hebbian (i.e., synapse-specific) and non-Hebbian (i.e., neuron-wide) mechanisms of LTP integrate information processing [77]. Indeed, one of the most thought-provoking hypotheses recently put forward is that genome-wide epigenetic changes may bias neurons towards cell-wide thresholds or set points, consequently modifying their susceptibility to Hebbian plasticity mechanisms and finally orchestrating a neuron's global response to the variety of molecular events involved in synaptic plasticity, suggesting a role in plasticity regulation and homeostasis [8]. The challenge is to functionally validate the relevance of specific epigenetic axes in learning and memory.

The amount of information obtained from cellular and molecular neuroscience of cognitive processes is overwhelming. However, the connection between this information and mechanistic conclusions at the cognitive level relies on important assumptions and generalizations. For example, while several molecular events in neurons signal plasticity mechanisms, the link between these mechanisms and the formation and loss of memories is only correlational. Even so, to verify whether the studied mechanisms are involved in cognition we still use behavioural tests in normal and diseased animal models. Thus, critical aspects for the assessment of rodent models of IDDs and the study of the underlying molecular mechanisms are face and predictive validity of the tests [78, 79]. In relation to DS and FXS, some tests on the best characterized mouse models (Ts65Dn [80] and Fmr1 KO [81]) stand out as widely accepted and relevant. In relation to long-term memory acquisition, consolidation, and retrieval, impaired in both syndromes, fear conditioning tests [82] and the Morris water maze [83] in combination with pharmacological interventions have shown face and predictive validity for both syndromes [84, 85]. However, construct validity and differences between the underlying biological causes of the syndromes are not well understood. To this end, more disease specific tests that provide experimental tools for preclinical therapeutic studies are required. In this regard, the development of behavioural paradigms, such as the touch-screen based tasks, can reproduce in mice the paradigms of The Cambridge Neuropsychological Test Automated Battery* (CANTAB)*, originally developed at the University of Cambridge in the 1980s [86]. Deepening on construct validity by the accurate assessment of cognitive domains provided by CANTAB-based touch-screen tasks in mice will allow not only improving the treatments of the syndromes, but also better understanding the biological substrates of cognition.

### 2.2. IDDs by Direct Mutation of Epigenetic Genes

**Figure 2:**
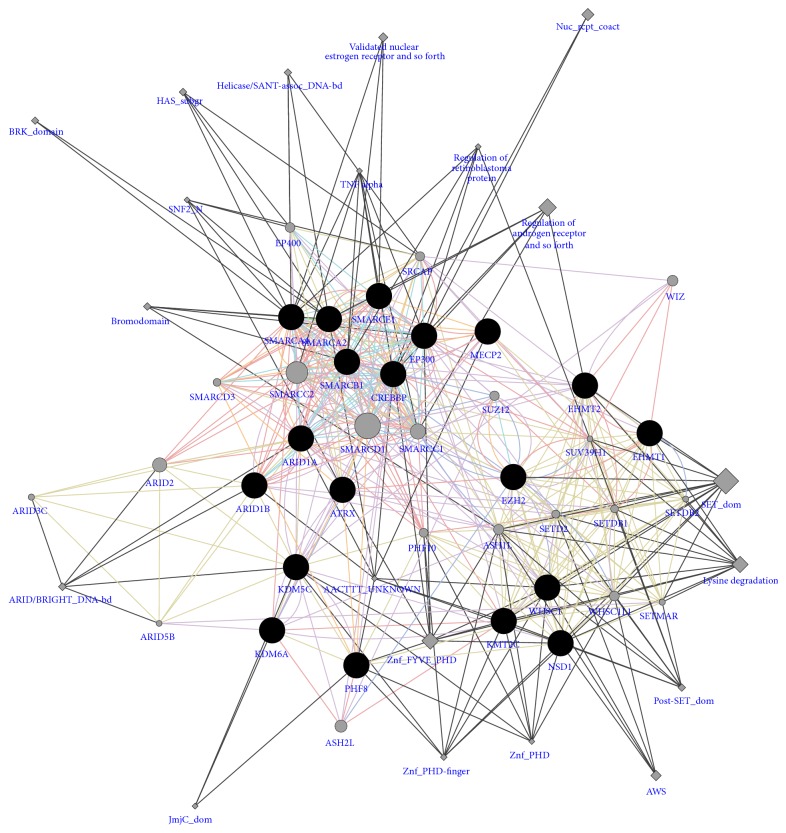
Protein-protein interactions of key epigenetic genes involved in genetic IDDs. Network of interactions of genes involved in the genetic IDDs arising from mutations in the epigenetic machinery (black nodes). Nodes sizes are related to the degree of the nodes (connectivity). Edge colours indicate coexpression (violet), colocalization (blue), genetic interactions (green), pathway (cyan), physical interaction (pink), predicted (orange), and shared protein domain (beige).

In the last decade, the discovery of mutations in the various components of the epigenetic machinery (writers, erasers, readers, and remodellers) has been linked to a number of well-known causes of IDDs [43, 87]. Intellectual disability is generally defined as deficits of intellectual function and adaptive behaviour that occur during the developmental period (see, e.g., http://aaidd.org/) and epigenetic disturbances are expected to have widespread downstream consequences (Figure 2). Rett Syndrome (RTT) is one of the most studied of such disorders, an X-linked dominant neurodevelopmental disorders, arising from mutations in a DNA methylation reader: the methyl-DNA-binding protein MeCP2. RTT patients show, next to morphological defects, a progressive cognitive impairment, autistic behaviour, and language and social impairments probably due to dendritic and spine atrophy [25]. MeCP2 normally results in transcriptional repression due to binding to methylated CpG (mCG) or CpA (mCA) dinucleotides, followed by HDAC recruitment [88]. However, MeCP2 can also result in transcriptional activation when binding to the promoters of some genes in association with the transcriptional activator CREB1 [89]. For instance, MeCP2 regulates the activity-dependent gene BDNF, keeping it switched off in absence of neuronal activity. Upon brain activity, MeCP2 gets phosphorylated and is released from BDNF promoter, enabling its expression [90]. Interestingly, it has been shown that longer genes have specific functions in the nervous system and tend to have a higher density of mCA. As a consequence, these genes are the most upregulated by MeCP2 knockout.

Another IDD directly arising from mutations in epigenetic players is Rubinstein-Taybi syndrome (RTS). Most of the RTS patients have mutations in the gene encoding for the cyclic AMP-responsive element binding protein (CREB) binding protein (CBP) [32], while in a minority of cases the mutations are in the gene encoding for p300 [33]. CBP and p300 are transcriptional coactivators with HAT activity, involved in development and cognition [91]. Interestingly, mouse model of RTS (CBP +/− mice) shows defects in synaptic plasticity due to impaired late phase long-term potentiation, with consequent defects in long-term memory. At the epigenetic level these mice show decreased histone acetylation that can be reversed by HDAC inhibition ameliorating the phenotype [92].

Several HMTs have been associated with congenital IDDs. A deletion containing the GLP/EHMT1 gene (euchromatin histone methyltransferase 1) causes Kleefstra syndrome, a developmental severe IDD, with defects in learning, motivation, and environmental adaptation. GLP/G9a is essential for regulating H3K9 dimethylation levels and regulates brain function through maintenance of the transcriptional homeostasis in adult neurons [37]. In minor cases Kleefstra syndrome is due to a de novo point mutation in the MLL3 gene, encoding for a H4K4 HMT [38]. Impaired H3K36 methylation is observed in two learning disabilities: Sotos Syndrome, due to NSD1 deletion [39], and Wolf-Hirschhorn syndrome, due to NSD2 deletion [40]. Mutation for the MLL2 gene, with reduced H3K4 methylation, is responsible for the Kabuki syndrome 1, with impaired hippocampus-dependent memory and developmental disorders [41], while de novo mutation at the EZH2 gene results in Weaver Syndrome 2, with impaired H3K27 methylation and consequent defects in neural differentiation [43]. Similarly, mutations in histone demethylases result in IDDs. Impaired H3K4me2/3 demethylation due to mutation in the KDM5C gene causes an autistic disorder called Claes-Jensen-type syndromic X-linked ID, with impaired brain development and plasticity [44]. Finally, mutations in the gene encoding the H3K9 demethylase PHF8 account for Siderius X-linked ID syndrome [44], while mutation in the gene KDM6A, H3K9 demethylases, gives Kabuki syndrome 2, with very similar clinical picture to Kabuki syndrome 1 [42].

Several mutations have been described in subunits of the nuclear remodelling complex nBAF, which are linked to IDDs and autism spectrum disorders (ASDs) [50]. The most affected genes belong to the SMARC and ARID families, the first having helicase and ATPase activity, the latter conferring DNA recognition binding sites. Examples of these IDDs are Coffin-Siris Syndrome (CSS) [47] and the Nicolaides-Baraitser syndrome (NBS) [48]. Another example of IDDs arising from mutation in chromatin remodelling components is the X-linked form of syndromic mental retardation associated with alpha thalassemia (ATRX syndrome), caused by point mutations in the ATRX gene, SWI/SNF chromatin remodelling containing an ATPase/helicase domain. These mutations have been shown to cause diverse changes in the pattern of DNA methylation, which may provide a link between chromatin remodelling, DNA methylation, and gene expression in developmental processes [49].

Since affecting epigenetic mechanisms, these mutations would lead theoretically to the deregulation of a very broad and nonspecific set of genes; however they surprisingly give rise to well defined syndromes, suggesting that they conversely lead to specific dysregulation of key genes. However, all these IDDs share common clinical features, indicating that they share common molecular pathways, deregulated upon epigenetic imbalance, which could be targeted therapeutically.

Note that even though these IDDs arise from mutations/deletions in specific components of the epigenetic machinery, the common molecular phenotype is a global epigenetic imbalance, affecting several epigenetic mechanisms. Histone modification and/or DNA modifications always occur in concert, with nuclear remodelling complexes bringing various epigenetic players at the regulatory regions of the genome. Moreover, several other disorders, even if not arising from direct impairment of the epigenetic machinery, show a strong epigenetic component, such as foetal alcohol spectrum disorders, neurodegenerative diseases (Alzheimer Disease, dementia, and Parkinson disease), poly-Q disorders (Huntington disease, spinal and bulbar muscular atrophy, and spinocerebellar ataxia type 3), autism spectrum disorders (ASDs), addiction, schizophrenia, stress, and Friedrich ataxia [91, 93, 94]. This suggests that epigenetics plays an important role in all neurological disorders characterized by defects in neurodevelopment and/or cognition. The establishment of a proper epigenetic balance could be the key in the treatment of these disorders.

### 2.3. Down Syndrome: A Global Epigenetic Perturbation

**Figure 3:**
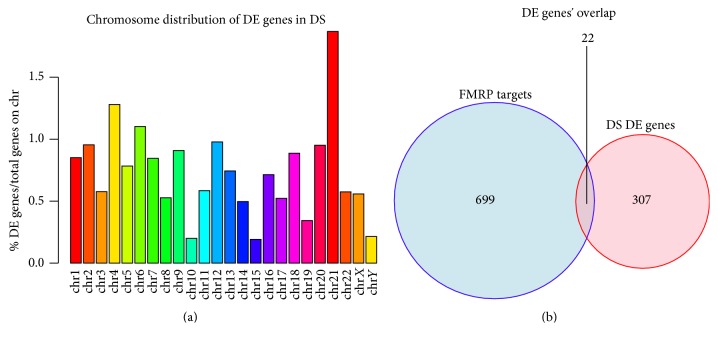
Differentially expressed (DE) genes in Down Syndrome (DS). (a) Bar plot showing the percentage of differentially expressed (DE) genes in DS over the total genes of each chromosome. (b) Venn Diagram showing the overlap among FMRP targets and a list of genes consistently expressed differentially in DS.

Down Syndrome (DS) is the most common genetic intellectual disability arising from the total of partial trisomy of chromosome 21, leading to a developmental disorder characterized by various defects, including impairments in language, memory, learning, and a higher frequency of developing Alzheimer Disease (AD) [1]. While DS would theoretically lead to 1.5-fold upregulation of all HSA21 genes, transcriptomic studies revealed that genes were differentially expressed on all chromosomes forming the so-called gene expression dysregulation domains (GEDDs), pattern of chromosome regions showing up- or downregulation of transcription in the trisomic cell along the whole genome. Interestingly, in DS actively transcribed regions are less expressed, while lowly transcribed regions are more expressed, leading to “flattening” of gene expression profiles. Further analysis of these data (GSE55504 [95]) has shown that even though the trisomic chromosome shows the highest fraction of deregulated genes, DS genes are distributed among all chromosomes (Figure 3(a), Ilario De Toma).

Epigenetic deregulation due to triplicated genes could explain the genome-wide change of gene expression, as chromosome 21 contains genes regulating all epigenetic aspects discussed in Section 1, and their overexpression due to the trisomy would easily affect the epigenetic balance, as will be reviewed in the following paragraphs.

#### 2.3.1. DNA Chemical Modifications in DS

DNMT3L is encoded on chromosome 21 and stimulates the activity of DNMT3a and DNMT3b [96]. Several studies have shown a deregulation in DNA methylation patterns in DS individuals, with a genome-wide hypermethylation [97–99], probably due to DNMT3L overexpression [100]. Some of the differentially methylated genes actually correlated with the cognitive impairment level in DS patients, such as TSC2, which has also been associated with the tau pathology in Alzheimer Disease [99]. The same widespread DNA hypermethylation was observed also in DS placenta, underlining the importance of epigenetic balance already at the foetal stage [101]. Conversely to the genomic hypermethylation, mitochondrial hypomethylation was seen in DS, probably due to reduced levels of the methyl donor SAM, leading to mitochondrial dysfunction [102]. Interestingly, mitochondrial dysfunction might affect histone modifications since the mitochondria are the source of high energy intermediates which are necessary for histone acetylation, deacetylation, methylation, and phosphorylation [103]. Finally, TET proteins are downregulated in DS by DNA methylation of the promoter of the genes from which they are encoded [97], resulting in a decrease in 5hmC levels and genomic hypermethylation [101].

#### 2.3.2. Histone Modifications and Chromatin Remodelling in DS

Many HSA21 genes influence specific histone modifications. An example is the phosphotyrosine kinase DYRK1A, which is able to regulate several proteins involved in epigenetic mechanisms. It promotes both histone deacetylation by phosphorylating SIRT1 [104] and histone acetylation by phosphorylating the CREB transcription factor, resulting in its binding with the HAT CBP [105]. DYRK1A also interferes with chromatin remodelling by binding nBAF and reducing the levels of the NRSF/REST neuron-restrictive silencing factor which is essential for neural differentiation [106]. Other HSA21 genes regulate the same key proteins: ETS2 [107] and the constitutive chromatin protein HMGN1 [108] influence the activity of CBP enhancing the H3K14 activity, while the activity of nBAF is modulated also by BRWD1 [109], a bromodomain containing protein recruiting nBAF to acetylated histones, and RUNX1 [110], which forms complexes that are associated with the active mark H3K4me3 and H4 acetylation. Moreover, HMGN1 not only interferes with histone acetylation, but inhibits phosphorylation of H3S10 and H3S28 [108] and inhibits the methyl binding protein MeCP2 by modifying the chromatin structure at the level of its promoter [111]. Finally, HSA21 encodes for two histone pseudogenes (H2AFZP and H2BFS) whose roles have not been elucidated yet, and the chromatin assembly factor 1B (CHAF1B) that forms a complex with the methyl-CpG binding protein MBD1 and the heterochromatin protein HP1 to favour chromatin repression through 5mC and H3K9me3 [112].

#### 2.3.3. ncRNAs in DS

HSA21 encodes for five miRNAs: mir-99a, mir125b2, mir155, mir802, and let-7c. Interestingly, mir155 and mir802 downregulate the methyl binding protein MeCP2 [113]. Furthermore, mir155 is also involved in synaptic dysfunction since it results in the downregulation of SNX27, a key component in the endosomal pathway that assures the glutamate receptor recycling [114]. Of note, mir125b levels increase also in AD brains [115]. Thus, besides the well-known role of APP overexpression, epigenetics could directly link AD and DS since a disturbance of epigenetic balance has also been observed in AD [116], partly explaining the higher frequency of early-onset AD in DS patients [1]. As regards long noncoding RNAs they constitute almost 35% of HSA21 annotated genes (GRCh38 assembly), making HSA21 the second chromosome with the highest percentage of long noncoding RNA after HSA18 (Ilario De Toma, personal communication). Future studies are needed to elucidate the role in epigenetics and cognition of these long noncoding RNAs [117].

Summing up, even though DS is caused by a precise genetic defect (trisomy 21), epigenetic mechanisms are globally dysregulated through various mechanisms. One of the main problems for obtaining a complete picture of epigenetic contributions in DS is that different studies performed analyses on different cell types and tissues, in different developmental stages (embryonic fibroblasts, neurons, blood cells, etc.). Since epigenetics is involved in differentiation and cell fate, this has led to different results that are often difficult to compare, as the epigenetic differences related to development and cell differentiation could mask the differences due to the trisomy.

### 2.4. FXS: Not Only Local Epigenetic Perturbation

Fragile X Syndrome (FXS) is the most common monogenic cause of intellectual disability, where a “CGG” triplet expansion at the 5′-UTR of the FMR1 gene is responsible for the loss of the Fragile X mental retardation protein (FMRP), a synaptically expressed RNA-binding protein regulating translation [2]. FMRP acts on its RNA targets in various ways: it influences RNAs stability, preventing or sustaining mRNA decay [118]; it transports RNAs from the cell body to synapses [119]; and it inhibits mRNA translation both by stalling ribosomes on their target mRNAs [2] and by inhibiting translation initiation [120].

When the CGG repeat expands between 56 and 200 (permutation), the FMR1 gene is upregulated with increased histone acetylation in the promoter [121], while in full mutation patients (>200 repeats) the FMR1 locus is transcriptionally repressed through cytosine methylation directed towards the repeats and the nearby sites constituting the CpG island. This results in demethylation and deacetylation of H3K4, methylation of H3K9 and H4K20, and trimethylation of H3K27 [122], with final transcriptional repression of the whole region. The failure in the heterochromatinization of the FMR1 locus in subjects with over 200 repeats translates in the complete lack of penetrance of the syndrome. These healthy carriers are called unmethylated full mutation (UFM) carriers and have a normal epigenetic profile (with the exception of partial H3K9 methylation), with a 30–40% increase in FMRP levels (as in permutation carriers) [123].

Interestingly, DNA demethylation with demethylating agents such as 5-azadeoxycytidine (5-azadC) reactivates FMR1 transcription in full mutation patients, with restoration of euchromatic marks (H3K4 methylation and acetylation) and partial reduction of the repressive H3K9 methylation [124, 125]. Even though 5-azadC was enough to reactivate the FMR1 locus, costimulation with HDAC inhibitors revealed synergic effect, yet ineffective alone [124]. However, inhibiting specifically the class III HDAD SIRT1 is effective in reactivating the FMR1 locus with an increase in H3K9 and H4K16 acetylation, while leaving unaltered DNA methylation. Since DNA demethylation leads to acetylation of H4K16 but not H3K9, it could be that H3K9 deacetylation is an early event, which is followed by DNA methylation and H4K16 deacetylation [126]. Similarly to some other long noncoding RNAs, the FMR1 transcript plays a direct role in gene silencing by directing the recruitment of repressive complexes like PRC2 to the locus, with consequent histone H3K27 methylation. This could be important in the beginning of the process of FMR1 repression [122]. The mechanism is still not known but could involve R loops made by the FMR1 transcript, particular conformations due to the repeat expansion [127].

One of the most debated questions is if this epigenetic deregulation has a genome-wide effect or affects only the FMR1 locus. A recent work succeeded in detecting differential DNA methylation only at the FMR1 locus, which is wholly affected. However, the study does not discriminate DNA methylation and hydroxyl-methylation (which are often diametrically regulated) and used the HumanMethylation450 BeadChip kit, taking into account just a specific subset of the genome [128].

Indeed, a global deregulation is possible since a lot of FMRP target mRNAs are involved in chromatin remodelling such as* HDAC4/5*,* NCOR1*–*3*, and* CBP* [2] and several ncRNAs [129, 130], whose transcript and protein levels are presumably altered by FRMP absence. Moreover, in the complex FMR1 locus, several ncRNAs are encoded, but most of them have not been characterized yet. One of these ncRNAs is FMR4, which is switched off similarly to FMR1 in full-length expansions. This lncRNA regulates target genes at distal locations such as the methyl-CpG-binding domain protein 4 (MBD4), hampering neural differentiation in FXS [131]. Interestingly, FMRP target genes are enriched in long genes and significantly overlap with MeCP2-repressed genes. As we said these genes are enriched in mCA and are important for brain function [90]. Once again this is emblematic of the molecular pathway commonalities across IDDs involving epigenetic mechanisms (in this case FXS and RTT).

FMRP has been shown to be involved in dendritic mRNA localization, synaptic protein synthesis, and synaptic plasticity. The mechanism relies on mGluR signalling in glutamatergic postsynaptic sites. When mGluR channels are active in a synapse, a phosphorylation cascade is triggered that affects the LTP pathway and triggers rapid local protein synthesis of preexisting dendritic mRNAs, including FMRP, around the active synapse [132]. As a result of FMRP regulation, proper tuning of the translation dynamics involved in mGluR-dependent LTD is established in active synapses. Even though the mechanism of dendritic spine maturation is not fully elucidated, recent observations suggest that the proper pruning and maturation of synaptic spines (impaired in FXS) rely on the interplay between local dendritic BDNF mRNA translation and secretion, with FMRP playing a key role in the regulation of these local events [133]. How the inactivation of FMRP and its effects on local translation interact with actin polymerization, or proteins such as cofilin, myosin, Arp2/3, and profilin [134] is an open question.

### 2.5. DS and FXS, Differences and Similarities

DS and FXS show striking similarities and differences. Both intellectual disabilities are common genetic developmental disorders characterized by specific defects in structural and synaptic plasticity due to alterations in specific molecular pathways. However, those alterations are often opposite, with the common final outcome of cognitive impairment [135]. DS patients show reduced dendritic branching and complexity in pyramidal neurons along with fewer and abnormal spines with enlarged heads that could explain the cognitive deficits [5]. This goes along, at the molecular level, with alterations in synaptic plasticity molecular pathways: long-term potentiation (LTP), the ability of the neuron to strengthen its synapses, is suppressed in DS mouse models [136], while long-term depression (LTD), the ability to weaken unused synapses, is enhanced [137]. Conversely, in FXS patients the cognitive impairment goes along with an increased density of thin and elongated spines in the same neurons [138]. Looking at the regulation of molecular pathways in FXS, while the role of LTP is controversial [139, 140], LTD is strongly induced, due to the overactivation of glutamate receptors [132].

Although presenting opposite phenotypes, DS and FXS share defects in dendritic spine morphology due to alterations in local protein synthesis. Both HSA21 RCAN1 and FMRP regulate calcineurin (CaN) activation, which is important for cofilin dephosphorylation. RCAN1 normally keeps calcineurin inactive; this increases phosphorylated cofilin that facilitates actin polymerization at the spine level. The enlarged spine heads observed in DS patient are probably due to RCAN1 overexpression. As a matter of fact, RCAN1-overexpressing mice show a phenotype similar to DS, with reduced volume and neuron number in the hippocampus, defective neurogenesis, enlarged spine heads, enhanced local protein synthesis of dendra, and impaired LTP [141, 142]. On the contrary in FXS patients, the silencing of the FMR1 locus results in the increase of the FMRP target PP2AC [143], phosphatase that dephosphorylates cofilin. This leads to the formation of long and thin filopodia-like spine heads, hallmark of FXS, due to defective actin polymerization. Calcineurin activates also local protein synthesis by dephosphorylating FMRP and allowing in this way the translation of the FMRP targets required for local protein synthesis and synaptic plasticity such as *α*CaMKII [142]. The ability of RCAN1 to bind and inhibit CaN is modulated also by the HSA21 gene DYRK1A, a serine threonine kinase important for synaptogenesis and spine actin dynamics [144]. This further links DS and FXS deregulation in the pathway of local protein synthesis.

Recent analyses from our group (Ilario De Toma, personal communication) show the link between DS deregulated molecular pathways and affected proteins in FXS by comparing 324 genes found to be consistently deregulated in DS in a published meta-analysis [145], with a list of FMRP targets [2]. The overlap was extremely significant (*p* < 0.0005, hypergeometric test) and included 9 HSA21 genes. Among those genes,* APP* is involved in Alzheimer Disease, which as we already stated has an early onset in DS patients [146, 147];* SYNJ1/synaptojanin* regulates neurotransmission together with two other HSA21 genes, intersectin/DAP160 and RCAN1 [148], and is involved in learning and memory;* Tiam1* and* Ttc3* are involved in neurogenesis [149]; and NRIP1 is needed for cognition and recruits HDACs [150] (Figure 3(b)). Finally, even though not present in our list of genes consistently deregulated in DS, the HSA21 gene DSCAM is a FMRP target and is involved in neural development [151].

One interesting question that needs to be unravelled is whether the epigenetic deregulation is upstream of the deregulation of those molecular pathways. This would allow a common therapy for both disorders to rescue the epigenetic imbalance at the base of their aetiology.

## 3. Restoring a Balanced Epigenetic State for the Treatment of ID

Historically the treatment of DS and FXS has focused on restoring the neurotransmitter balance that is compromised in the two disorders or on replacing deficits in different systems. As mentioned before, in FXS there is global hyperexcitation due to overactivation of the glutamatergic pathway, while in DS there is an overinhibition due to the predominance of the GABA inhibitory pathway. Therefore in the attempt to restore the neurotransmitter balance, agonist and antagonist for both glutamate and GABA receptors have undergone clinical trials. However results have been unsuccessful by now, due to lack of efficacy and or safety [152, 153]. For instance, inhibiting the GABA pathway in DS may increase the susceptibility of DS patients to epileptic seizures, together with side effects in various developmental processes [154].

In the US, commercial formulations aimed at ameliorating the DS phenotype are composed mainly of antioxidant and folates. The rationale behind this is that DS patients overexpress two HSA21 encoded enzymes, SOD-1, leading to an increase in reactive oxygen species production, and cystathionine *β*-synthase, resulting in folate deficiency. However clinical trials showed that this approach is ineffective [155].

**Figure 4:**
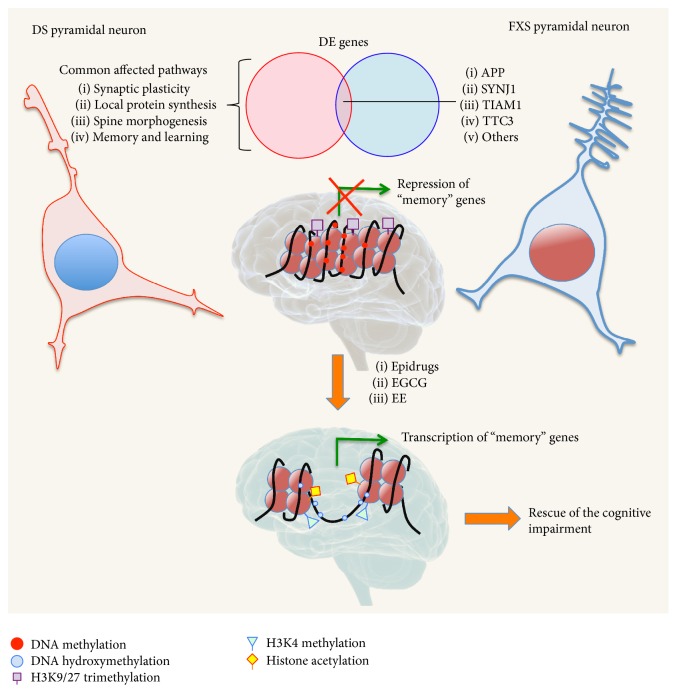
Reestablishing a balanced epigenetic state to rescue the cognitive impairment in Down Syndrome (DS) and Fragile X Syndrome (FXS). Cartoon representation of a DS (left) and FXS (right) pyramidal neuron. In both syndromes alterations in synaptic plasticity, local protein synthesis, spine morphogenesis, and memory and learning contribute to the cognitive impairment. However the structural phenotype is distinct: DS neurons have large and* stubby* spines, while FXS neurons have long immature filopodia-like spines. Key genes involved in these pathways (e.g., APP, SYNJ1, TIAM1, and TTC3) are commonly deregulated due to epigenetic modifications at the chromatin level. The cartoon shows the repression of “memory” genes by DNA methylation (red circles) and H3K9 and H3K27 trimethylation (violet squares) in DS and FXS, with consequent chromatin compaction. Conversely, epigallocatechin gallate (EGCG) and Environmental Enrichment (EE) or directly* epidrugs* can reactivate the chromatin state at the level of “memory” gene by DNA hydroxymethylation (blue circles), H3K4 methylation (green triangles), and histone acetylation (yellow squares), rescuing the cognitive deficits.

None of these traditional approaches have been revealed as safe and effective in the treatment of IDs. However, a possible future therapy based on the direct or indirect modulation of epigenetic mechanisms is promising. Restoring a balanced epigenetic state will be key to renormalize the altered expression in master regulator genes involved in the cognitive problems (Figure 4).

### 3.1. Environmental Enrichment: An “Epigenetic” Treatment

As we have previously pointed out in this review, the environment is a main driver of epigenetic modifications. During development, the microenvironment allows the genome to be interpreted differently by different cell types and in different developmental stages and contexts. The effect of the environment on gene expression is particularly evident in the case of monozygotic twins that are genetically identical but phenotypically and epigenetically different, especially when grown apart [156]. Environmental Enrichment (EE) is an effective protocol used in rodent models to boost learning and memory. The paradigm consists in keeping laboratory mice in a so-called enriched environment with respect to laboratory standards: larger cages, larger groups, various stimulatory objects such as toys of all sort, and running wheels. The aim is to provide the animals every kind of sensory, cognitive, and motor stimuli such as the possibility to establish more complex social interactions, to explore and play with new objects, and the opportunity for voluntary physical activity. Interestingly, EE improves learning and memory, enhancing long-term potentiation [157], and delays or rescues deficits in a variety of mouse models of neurological disorders [158]. Of note, EE is effective in both FXS and DS models. In* Fmr1* KO mice, EE rescued behavioural and neuronal abnormalities, activating the glutamatergic signalling and increasing dendritic branching, spine number, and maturation. Interestingly, EE acts independently of FMRP expression in Fmr1 KO mice, as it did not affect FMRP levels [159], but translates in reduced FMRP protein in mouse model of fragile X premutation [160]. Similarly, EE protocols increased dendritic branching and spines in DS models [161], probably by normalizing DYRK1A levels [162]. Epigenetic mechanism could be involved in the effects of EE in IDDs, since EE-induced benefits are long-lasting (at least 3-4 weeks) and are supported by a specific EE-dependent transcriptional profile, which is likely activated through epigenetic mechanisms [163]. Four-week housing in EE conditions, while rescuing impaired memories in both contextual fear conditioning and water maze assays, was associated with enhanced histone acetylation on several residues. This effect was mimicked by daily injection of HDAC inhibitors in the murine peritoneum [164]. Another interesting experiment used mice deficient in CBP, a transcriptional coactivator with histone acetyltransferase activity. EE improved some defects in behaviour and cognition caused by CBP deficiency and promoted synaptic growth. However, its ability to enhance spatial navigation and pattern separation and to induce neurogenesis was severely compromised in absence of CBP, with attenuation of the transcriptional profile normally associated with EE due to decreased acetylation of the promoter regions of genes involved in cognition [165], suggesting that CBP contributes to EE ability to activate gene expression through histone acetylation.

Understanding the full epigenetic, genetic, and molecular mechanisms of Environmental Enrichment will guide the development of a new class of therapeutics called “enviromimetics” for the treatment of IDDs. Enviromimetics are compounds aimed to mimic the beneficial effect of EE on cognition. An important unanswered question is how EE results in mouse models relate to human living experience, since most humans already do experience high levels of complexity and novelty in their natural environments. However, individuals vary widely for the kind and amount of mental exercise and physical activity performed. It will be extremely important in order to improve existing therapeutic approaches to closely reproduce in animal models the environmental factors relevant to human conditions [158]. Moreover research on EE paves the way for nonpharmacological treatment with promising outcomes in disorders such as DS and FXS if used in synergy with other cognitive enhancers.

### 3.2. Epigenetic Drugs in Intellectual Disabilities

It is now increasingly thought that approaches aimed at reestablishing a proper gene expression profile, especially of key genes impaired in cognitive disabilities, are the future of therapy [152, 153]. An efficient way to induce long-lasting transcriptional changes is to modulate epigenetic players, a field that is booming in cancer research [166]. Epigenetic changes are reversible and therefore are suitable to alleviate certain features in IDDs that originate from epigenetic alterations. Insights from cancer research could directly be conveyed to new “cognitive epigenetics.” As a matter of fact, FDA has approved four epidrugs against cancer, two DNMT inhibitors (5-azacytidine and decitabine) and two HDAC inhibitors [167]. Moreover valproic acid, which has already been used against epilepsy and bipolar disorders, has shown HDAC inhibitory and anticarcinogenic activity, being the first epidrug approved for neurological disorders [168]. One of the concerns related to the use of epidrugs is their potential genome-wide and nonchromatin effect, since, for example, HDACs can also act on nonhistone proteins. Although these unwanted effects are less severe than one might expect, a technology currently in development called “epigenetic editing” will allow specifically targeting epigenetic drugs to the gene(s) of interest, thanks to the usage of DNA binding domains such as zinc finger proteins [169].

### 3.3. Epigallocatechin-3-gallate (EGCG): Panacea for IDDs?

The flavonoid epigallocatechin-3-gallate (EGCG) is the most abundant polyphenol extracted from green tea. Strikingly, this molecule is effective in both mouse models of AD and DS. In AD mice, EGCG decreased beta-amyloid levels and plaques via ADAM10-mediated promotion of the alpha-secretase proteolytic pathway and modulates tau-profiles with final cognitive improvements [170]. Similarly, in DS models, EGCG recovered cognitive and neural plasticity phenotypes, a result that was replicated in a pilot clinical trial in humans [171]. Strikingly, a pilot clinical trial is undergoing for EGCG treatment of FXS individuals (https://clinicaltrials.gov/ct2/show/NCT01855971). EGCG has a plethora of different effects and has thus been investigated in studies from various research areas, including cancer research [172]. However, the heterogeneous effects make it difficult to fully identify and understand the underlying molecular therapeutic mechanisms. Among its properties, it has antioxidant and anti-inflammatory effects and is able to regulate several enzymes by modulating their kinase activity. Moreover, EGCG interferes at various levels with epigenetic mechanisms, affecting the chromatin state. EGCG inhibits both DNA methyltransferases [173] and class I histone deacetylases (HDAC 1, 2, 3, and 8) [174, 175]; it reduces the level of H327me3 and H2AK119 ubiquitination by reducing polycomb protein levels [176] and affects miRNAs expression [177]. The property of EGCG of modulating epigenetic changes makes it an ideal candidate for the treatment of IDDs including DS and FXS. Its widespread epigenetic effect might reestablish the lost epigenetic balance, acting in a context specific way and resulting in being effective in several IDDs, even if the source and kind of epigenetic dysregulation are different. Many properties of EGCG would contribute to its efficacy. For instance, besides its epigenetic effect, EGCG inhibition of DYRK1A kinase activity results in normalization of this gene which is crucial for DS pathology [178]. In addition, EGCG could also act by rescuing DS mitochondrial dysfunction as it stimulates mitochondrial biogenesis and rescues oxidative phosphorylation [179]. Remarkably, DYRK1A is also involved in epigenetic regulation (see Section 2), suggesting that EGCG could both directly and indirectly regulate the epigenetic state. This interconnection is even stronger if we consider that, similarly to EGCG effect, enriched environments rescue defects in DS, normalize DYRK1A levels, and modulate epigenetic modifications. EGCG can thus be considered an “enviromimetics.” Of note, a recent study showed that the combination of EE and EGCG acts synergistically in ameliorating learning alterations and age-related cognitive decline in DS [180], underlining the potential of combinatorial therapeutic approaches.

## 4. Conclusion and Future Perspectives

Developmental disorders are often characterized by intellectual disability due to defects in structural and synaptic plasticity, with impaired activity-dependent cognitive-related molecular processes such as local protein synthesis, long-term potentiation, and long-term depression. In this context, it is difficult to discern what can still be rescued in cognitive developmental disorders and what is irreversibly lost. Epigenetics is not only indirectly needed for cognition by regulating neurodevelopment, but, as we amply discussed in this review, directly regulates experience-based cognitive processes. Epigenetics intercalates in development, cognition, and aging/neurodegeneration, playing a key regulatory role in all these processes. For instance, DNA methylation allows cells to be “programmed” and differentiate during development, is dynamically regulated during cognitive processes, and increases gradually with aging. In contrast to genetic alterations, epigenetic modifications are reversible and this gives a great therapeutic potential to epigenetic drugs to at least partially revert the phenotype associated with IDDs. We have reviewed how epigenetic treatments restore cognitive deficits in various models of cognitive impairment, restoring a correct balance among writers and erasers of epigenetic modification. Of course an early treatment will maximize the efficacy of epidrugs, since the more differentiated a tissue, the less reversible the phenotypes.

Two main concerns are associated with epigenetic treatment: genome-wide nonspecific effects and toxicity. As regards the genome-wide effects, it would be worth conceiving ways to deliver epigenetic drugs such as DNMTi and HDACi to the cell types that actually show the epigenetic imbalance. Moreover, the epigenetic editing approach might be a promising solution, allowing directing the epigenetic drug to the loci of the key genes involved in the IDD. Noticeably, the genome-wide action of epigenetic drugs may also be an advantage, since it makes it possible to restore the epigenetic balance in disorders such as DS and FXS, where a similar cognitive impairment is originated by different morphologic phenotypes affecting common altered synaptic plasticity pathways. In this case the same molecule will work in a context specific manner on different loci, since the “substrate” of their action (the syndromic chromatin state) will be different in both cases, restoring the impaired epigenetic balance.

However, the epigenetic reversibility property is a double-edged sword. Since epigenetic changes are reversible, some epigenetic drug formulations are simply not long lasting. That would, for example, account for what has been found in the pilot clinical trial involving EGCG and the rescue of the cognitive impairment in DS patients, where stopping EGCG treatment leads to the reappearance of the impaired phenotype [171]. To overcome this problem the future of therapeutic treatment for cognitive disorders should focus in potentiating and extending the effect of epidrugs, while at the same time reducing the toxicity associated with chronic treatment. In this sense, combinatorial therapy could play an important role, having synergic effect as it has been shown in mouse models for EE and EGCG [180] or in the synergic effect of DNA demethylation and histone hyperacetylation in the reactivation of the FMR1 gene in human cell cultures [124]. Moreover this approach could be combined to conventional treatments, such as neuromodulators aimed at restoring the neurotransmitter balance in DS and FXS. Tackling the epigenetic deregulation from many sides, together with the targeting of specific molecular pathways, will allow both to reduce the dose and thus the toxicity of the drugs in the formulation and to extend their efficacy.

To this end a deeper understanding of all the epigenetic, transcriptional, and molecular cascades activating upon cognition in both physiological and pathological contexts is needed. Future integrative studies will combine epigenetic data, transcriptional data, and molecular data to get new insights into the pathogenesis of IDDs, focusing both on common altered pathways and to specific mechanisms. The development of new technologies and the increase in high-throughput data will allow in a near future elucidating the cognitive processes that are dysregulated in IDDs. Most has yet to come. For instance, as regards DNA methylation, one of the first epigenetic modifications that has been studied, it would be important to discriminate among 5mC and 5hmC, since both are highly present in the adult brain and have opposite outcomes on transcriptional regulation. A new technique called TET Assisted Bisulfite sequencing (TAB-seq) in conjunction with bisulfite sequencing, allows discerning between 5mC and 5hmC at single base resolution [181]; however very few datasets of this kind are available by now due to cost-related issues.

The main problem with previous studies is that they lack cell-specificity. For example, many studies on DS focus on different types of cells (e.g., blood cell, fibroblast) and different animal models, taking cells at different developmental stages, with results that are difficult to compare. Moreover, the brain is probably the most complicated organ, where several different cell types such as excitatory pyramidal cells, inhibitory interneurons, and glial cells compose even a reasonably delimited part, such as the hippocampus. Since epigenetic changes are responsible for cell fate, the epigenetic variations associated with cell type determination will sum up to the epigenetic changes associated with the syndromic state or to the cognitive processes, and this will dramatically reduce the power of the studies. Differences in cell type composition in compared samples of complex tissues will result in difficulties to distinguish treatment or disease specific changes from “epigenetic noise” caused by cell type specific marks varying based on cell type fractions. Therefore, cell type specific studies using techniques such as cell sorting to focus on single cell populations will have an increased power in detecting epigenetic differences and underpinning new key mechanism of regulation.

We speculate that epigenetic drugs, such as EGCG in combination with other cognitive enhancers and specific drugs interfering with the cell and disorder specific molecular targets, will allow the recovery of the epigenetic balance lost in IDDs such as DS and FXS, making the healing of the cognitive impairment possible.

## Figures and Tables

**Table 1:** Epigenetic mechanisms in cognition and IDDs.

Epigenetic changes	Writers	Erasers	Readers	Effect on gene expression	Effects on cognition	IDDs involved
5mC	DNMTs	TETs AID/Apobec	MBPs	Repression	Memory formation [17, 18] Memory consolidation [24]	Rett Syndrome [25]

mCH	DNMT3a	TETs AID/Apobec	MBPs (e.g., MeCP2)	Repression		Rett Syndrome [26]

5hmC	TETs			Activation	Memory formation [22] Memory extinction [23] LTD [23]	

Histone acetylation	HATs	HDACs (HDAC1–5, SIRT1)	CPB BRDs	Activation	Memory formation [27] Memory consolidation [28, 29] Depression [30] Fear extinction [31]	Rubinstein-Taybi syndrome [32, 33]

Histone methylation	HMTs (MLL, GLP/G9a)	HDMs (LSD1, JMJD1a)	Chromodomains (e.g., HP1)	Activation/repression	Memory formation [34] Memory consolidation [35] Adaptive behaviour [36]	Kleefstra syndrome [37, 38] Sotos syndrome [39] Wolf-Hirschhorn syndrome [40] Kabuki syndrome [41, 42] Weaver syndrome 2 [43] Claes-Jensen-type syndromic X-linked ID [44] Siderius X-linked ID [45]

Chromatin remodelling			nBAF, ATRX	Activation/repression	Long-term memory formation [46]	Coffin-Siris Syndrome [47] Nicolaides-Baraitser syndrome [48] ATRX syndrome [49] ASD [50]
